# Supplementary material for: Deep profiling of multiple ischemic lesions in a large, multi-center cohort: Frequency, spatial distribution, and associations to clinical characteristics
Source: Front Neurosci. 2022 Aug 25;16:994458. doi: 10.3389/fnins.2022.994458 (PMC9453031; doi:10.3389/fnins.2022.994458)
Supplement: Supplementary file 1 [file Data_Sheet_1.docx]

**Supplemental materials**

**Methods:**

**Neuroimaging parameters**

Neuroimages were recorded with 1T, 1.5T or 3T scanners (General Electric Medical Systems, Philips Medical Systems, Siemens, Toshiba, Marconi Medical Systems, Picker International, Inc.).

**Diffusion-weighted images (DWI):** Mostly axial orientation (2727/2770 axial, 43/2770 coronal). Axial: Reconstruction matrix 256x256mm^2^ (range: 128x128mm^2^ to 432x384mm^2^), median field-of-view 230 mm (range: 200 to 420 mm), median slice thickness 5mm (range: 2 to 7mm, gaps of 0 to 3mm), median TR 4.773ms, median TE 92ms. Coronal: reconstruction matrix 256x256 mm^2^, median field-of-view 260mm, median slice thickness 5mm, median TR 8.200ms, median TE 112ms. In most cases 3 directions (range: 3 to 25). In most cases low b-value 0s/mm^2^ (range: 0 to 50s/mm^2^), high b-value 1000s/mm^2^ (range: 800 to 2000s/mm^2^).

**Model specification for stroke severity**

**Hyperpriors**

*σ_ β* ∼ *Halfcauchy(1)*

*mu_ β* ∼ *Normal(µ = 0, σ = 10)*

**Priors**

*⍺* ∼ *Normal(µ = 0, σ = 20)*

*β_single, multiple_* ∼ *Normal(µ = mu_ β, σ = σ_ β) _single, multiple_*

**Likelihood**

*NIHSS_est = ⍺ + β [single/multiple]* lesion volume*

*eps* ∼ *Halfcauchy(20)*

*stroke_severity* ∼ *Normal(µ = NIHSS_est, σ = eps)*

**Results:**

**Bayesian hierarchical regression: Interaction effects of multiple lesions and lesion volume with respect to stroke severity.**

Findings remained broadly the same when analyzing patients with anterior circulation strokes and without excluding patients with lacunar lesions: Effect of lesion volume on stroke severity was similar for patients with a single and with multiple lesions (posterior distribution for single lesions: mean: 1.49, 90% HPDI: 1.33 to 1.66, posterior distribution for multiple lesions: mean: 1.51, 90% HPDI: 1.38 to 1.68; difference of posterior distributions: mean: -0.0144, 90% HDPI: -0.094 to 0.0485).
